# Structural Characterization of Black Widow Spider Dragline Silk Proteins CRP1 and CRP4

**DOI:** 10.3390/molecules25143212

**Published:** 2020-07-14

**Authors:** Mikayla Shanafelt, Taylor Rabara, Danielle MacArt, Caroline Williams, Ryan Hekman, Hyun Joo, Jerry Tsai, Craig Vierra

**Affiliations:** 1 Departments of Chemistry and Biological Sciences, University of the Pacific, Stockton, CA 95211, USA; shanafeltm1@gmail.com (M.S.); taylor.rabara@gmail.com (T.R.); d_macart@u.pacific.edu (D.M.); hjoo@pacific.edu (H.J.); jtsai@pacific.edu (J.T.); 2 Institute for Biomedical Science Center for Microbial Pathogenesis, Georgia State University, Decatur, GA 30302, USA; c_williams15@u.pacific.edu; 3 Center for Network Systems Biology, Boston University, Boston, MA 02215, USA; rhekman@bu.edu

**Keywords:** spider silk, dragline silk, spidroins, synthetic silk, black widow spider

## Abstract

Spider dragline silk represents a biomaterial with outstanding mechanical properties, possessing high-tensile strength and toughness. In black widows at least eight different proteins have been identified as constituents of dragline silk. These represent major ampullate spidroins MaSp1, MaSp2, MaSp’, and several low-molecular weight cysteine-rich protein (CRP) family members, including CRP1, CRP2, and CRP4. Molecular modeling predicts that CRPs contain a cystine slipknot motif, but experimental evidence to support this assertion remains to be reported. To advance scientific knowledge regarding CRP function, we recombinantly expressed and purified CRP1 and CRP4 from bacteria and investigated their secondary structure using circular dichroism (CD) under different chemical and physical conditions. We demonstrate by far-UV CD spectroscopy that these proteins contain similar secondary structure, having substantial amounts of random coil conformation, followed by lower levels of beta sheet, alpha helical and beta turn structures. CRPs are thermally and pH stable; however, treatment with reagents that disrupt disulfide bonds impact their structural conformations. Cross-linking mass spectrometry (XL-MS) data also support computational models of CRP1. Taken together, the chemical and thermal stability of CRPs, the cross-linking data, coupled with the structural sensitivity to reducing agents, are experimentally consistent with the supposition CRPs are cystine slipknot proteins.

## 1. Introduction

Spider silk has extraordinary mechanical properties, including high tensile strength and toughness [1,2]. Spiders are capable of manufacturing 6–7 distinct silk types, each finely tuned to perform a diverse range of biological functions [3,4]. The most widely studied silk type in the scientific community is dragline silk. Dragline silk, also referred to as the “safety-line” of the spider, is extruded from the major ampullate (MA) gland, a biofactory residing in the abdomen of the spider. Histological analyses have shown that the MA gland consists of three regions: a tail, ampulla, and spinning duct [5,6]. Because dragline silk represents a high performance fiber that outperforms a vast majority of natural and man-made materials used in engineering, much effort has been focused on elucidating the constituents of dragline silk as well as the molecular mechanisms that control its pathway of silk extrusion [6,7].

Initially, dragline silk was described as a two-protein fiber, consisting of the spidroin family members, MaSp1, and MaSp2 [8,9]. Translation of the full-length DNA sequences for MaSp1 and MaSp2 demonstrate highly repetitive, Gly-Ala-rich large molecular weight proteins [10]. Protein architectures of MaSp1 and MaSp2 include a non-repetitive, conserved N- and C-terminal domain (NTD and CTD) and internal block repeats consisting of about 35–40 amino acids. The NTD and CTD have been implicated in controlling the silk assembly process, and specifically, the CTD has been reported to mediate solubility and storage [11,12,13]. The repetitive regions of spidroins contribute to the strength and extensibility of the fibers. Specifically, the repetitive segments of MaSp1 are dominated by Gly-Gly-X (GGX; X = A, Q, or Y), GA, and poly-A (4–10 consecutive Ala), whereas the MaSp2 amino acid sequence includes GPX (X = G or S), QQ, GGX (X is typically A), GSG, and poly-A (3–9 consecutive Ala) [10]. While MaSp1 and MaSp2 are stored in the ampulla, protein chain regions corresponding to the poly-A blocks facilitate beta sheet and nanocrystal formation, a structure that has been attributed to the high tensile strength of extruded fibers [6,14,15,16]. Given the superior mechanical properties of spider silk, but impracticability of farming arachnids on a large-scale format, there has been a concerted effort by scientists across the globe to manufacture synthetic spider silk for commercial applications. In order to achieve mass production of artificial fibers, investigators have focused their attention on two areas: 1) Development of heterologous expression systems capable of production of vast quantities of recombinant spidroins; 2) creation of spinning methodologies that recapitulate the extrusion process containing the natural constituents of the MA spinning dope [17,18,19].

Transcriptomic analyses of the cob-weaver spider *Latrodectus hesperus* (black widow) have shed light on highly expressed transcripts within silk-producing glands [20]. MaSp1, MaSp2, and MaSp’, along with 30 other gene transcripts were more abundantly expressed in MA glands relative to cephalothoraxes, suggesting additional potential candidates for involvement in spider silk assembly of dragline silk or constituents of the fibers themselves [20,21,22]. Further proteomic studies using mass spectrometry have led to the identification of non-MaSp proteins in spider silk [22,23]. These non-MaSp proteins include a family of low molecular weight cysteine-rich proteins referred to as CRPs. In black widow spiders, these encompass CRP1, CRP2, CRP3, CRP4, and CRP5 (CRPs) [23]. CRP1, CRP2, and CRP4 have been demonstrated to be constituents of the MA gland, spinning dope, and dragline silk fibers [24]. Because no quantitative proteomic studies have been reported for dragline silk, the abundance of the CRPs relative to the spidroin family members remains unclear. The presence of CRP1, CRP2, and CRP4 in dragline silk, however, suggests an important biological role in assembly of dragline silk and/or fiber mechanics. Forced dragline silking of female black widows leads to increased mRNA synthesis of CRP2 and CRP4, but a decrease in CRP1 mRNA levels, suggesting CRP1 potentially serves a distinct biological role relative to CRP2 and CRP4 [23]. Computational modeling of the primary sequences of CRP family members suggests these proteins fold into a cystine slipknot [23]. Proteins with cystine slipknot motifs have been identified as the strongest force clamps known in the protein world, suggesting CRPs potentially serve a mechanical role in dragline silk [25]. Cystine slipknots use disulfide bonds to hold the protein structure together. To date, the majority of studies have concentrated on the biophysical and mechanical properties of spidroins and little, if any, attention has been focused on CRP family members, despite their established presence in natural dragline silk.

In this study, we express and purify recombinant CRP1 and CRP4 from bacteria and investigate the impact of pH, temperature, as well as the importance of disulfide bonds in their secondary structure using circular dichroism (CD). Our data represent the first experimental findings that describe the solution state structure of CRP family members. They demonstrate recombinant CRP1 and CRP4 display high thermostability and stable structural conformations under pH and temperature conditions reported for proteins stored and extruded from the MA gland. We also show that treatment of recombinant CRP1 and CRP4 with reducing agents alters CRP secondary structure. This observation supports that disulfide bonds serve an important role in the structural conformation of recombinant CRP1 and CRP4, providing evidence to buttress the assertion that these proteins contain cystine slipknot motifs. Lastly, cross-linking mass spectrometry (XL-MS) studies with CRP1 and disuccinimidyl sulfoxide (DSSO), provide further experimental data that supports computational models of CRP1. Taken together, the elucidation of the in-solution structures of the CRPs is important to advance our understanding regarding the molecular mechanisms that govern spidroin assembly, silk extrusion, and fiber performance.

## 2. Results

### 2.1. Purification of Recombinantly Expressed CRPs from Bacteria

**Figure 1:**
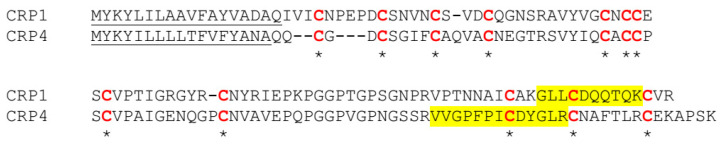
Amino acid sequence alignment of black widow CRP1 and CRP4. Single letter designations represent amino acid residues, while the underlined regions depict the secretion signal predicted by SignalP 4.1 (DTU Bioinformatics). Red letters or asterisks show conserved Cys (C) residues. Dashes represent an insertion to optimize protein alignments. Highlighted yellow regions correspond to select peptides identified by MS/MS analysis (see Figure 3).

MS/MS studies have demonstrated CRP1, CRP2, and CRP4 are constituents of dragline silk fibers, heightening the importance of elucidation of their function, especially since laboratories across the globe are racing to manufacture protein blends to spin synthetic silk for commercialization. Because CRP2 and CRP4 mRNA levels both increase when black widows are forcibly silked for dragline silk, while CRP1 mRNA levels decrease, we focused on the structural characterization of CRP1 and CRP4 because of their differences in transcriptional behavior upon silking and our ability to routinely detect these two proteins in the MA glands and dragline silk fibers by mass spectrometry. As shown below, CRP1 and CRP4 cDNAs encode low molecular weight proteins that are cysteine-rich, each containing 12 conserved cysteine residues (Figure 1).

**Figure 2:**
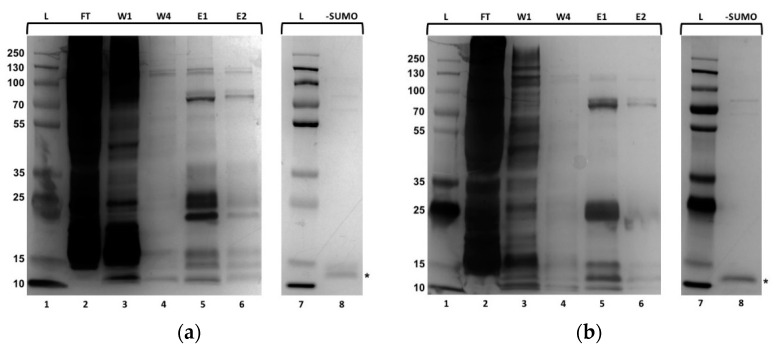
Isolation of recombinant black widow CRP1 and CRP4 from *E. coli* crude extract. SDS-PAGE analysis of purified CRP1 and CRP4 after affinity chromatography using a Ni-NTA agarose resin. Ubiquitin-like protease (ULP1) was used to remove the 10×His-SUMO tags from purified 10×His-SUMO-CRP1 and 10×His-SUMO-CRP4. Proteins were visualized using silver staining. FT = flow through; W1 and W4 = wash 1 and 4; E = elution 1 and 2; L = protein ladder in kDa; -SUMO = removal of tag. Asterisks represent cysteine-rich proteins (CRPs) lacking tags. (**a**) CRP1; (**b**) CRP4.

To study the physical and chemical properties of CRP family members, 10×His-tagged SUMO-CRP1 and CRP4 fusion proteins were expressed and purified from crude bacterial lysate using Ni-NTA affinity chromatography (Figure 2a,b). After collection of the flow through (FT), wash (W), and elution (E) fractions, protein samples were size-fractionated using SDS-PAGE analysis, followed by visualization using silver staining (Figure 2a,b, lanes 2–6). Analyses of CRP1 and CRP4 FT fractions revealed dark smearing patterns, likely because of the presence of endogenous bacterial proteins unable to bind the nickel resin (Figure 2a,b, lanes 2, respectively). Wash fraction one (W1) contained similar, but less prominent smearing patterns, while the last wash fraction (W4) appeared to lack protein, signifying non-specific bacterial proteins were removed from the resin (Figure 2a,b, lanes 3–4, respectively). The first two elution fractions (E1–2) revealed distinct protein bands migrating at approximately 25-kDa (Figure 2a,b, lanes 5–6, respectively). These experimental masses were consistent with predicted molecular masses of the 10×His-SUMO-CRP fusion proteins (10×His-SUMO and CRP1 or CRP4 molecular masses were approximately 15- and 10-kDa, respectively).

Because the 10×His-SUMO appendage could potentially influence secondary structural characteristics of CRP1 and CRP4, we employed a strategy to remove the 10×His-SUMO tag from the CRPs. Purified 10×His-SUMO-CRP1 and 10×His-SUMO-CRP4 were treated with recombinant ubiquitin-like-specific protease 1 (ULP1) to remove the 10×His-SUMO segment. After incubation of 10×His-SUMO-CRP1 or CRP4 with ULP1, His-tagged ULP1, 10×His-SUMO, and the CRP components were reapplied to a nickel-NTA agarose column, which allowed for capturing of the His-tagged ULP1 and 10×His-SUMO region. Analysis of the ULP1-cleaved flow through CRP1 and CRP4 samples by SDS-PAGE analysis, followed by silver staining, detected bands migrating at the expected mass range for CRP1 and CRP4 (Figure 2a,b, lanes 8 [-SUMO], respectively). An additional, slower migrating band was observed for CRP1; this species likely represents a partially unfolded form of CRP1 (Figure 2a, lane 8). 

**Figure 3:**
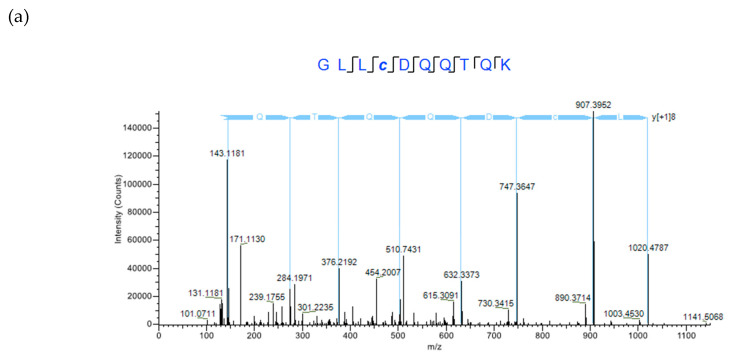

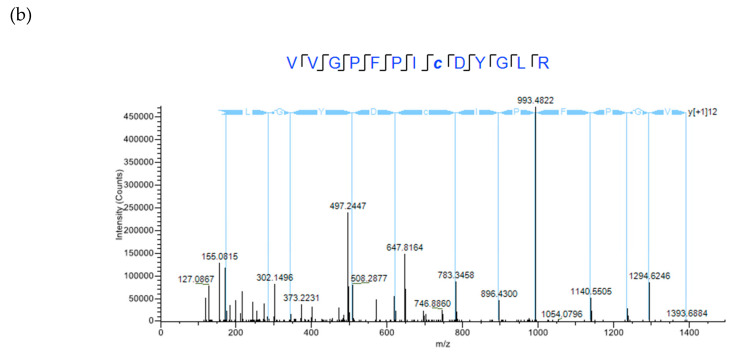
MS/MS spectra of purified CRP proteins after in-solution tryptic digestion. Samples were analyzed using an Orbitrap Fusion™ Tribrid™ mass spectrometer to confirm purified proteins corresponded to CRP1 and CRP4 lacking 10xHis-SUMO tags. (**a**) CRP1; (**b**) CRP4.

To confirm these bands represented CRP1 and CRP4, we performed in-solution tryptic digestions of the flow-through samples. Tryptic digests were then analyzed by an Orbitrap Fusion™ Tribrid™ mass spectrometer equipped with nano-HPLC. MS/MS data were acquired using high collision dissociation fragmentation and orbitrap detection. Two product ion spectra from peptides 1190.58 and 1492.76 ([M + 2H]^2+^) corresponded to peptide sequences GLLCDQQTQK and VVGPFPICDYGLR derived from CRP1 and CRP4, respectively (Figure 3a,b; also see Figure 1). Cysteine residues in both peptides were alkylated by iodoacetamide (IAA).

MS/MS analyses led to 100% and 94% sequence coverage for CRP1 and CRP4, respectively. No 10×His-SUMO or His-tagged ULP1 were detected in the flow through fraction, indicating that purified CRP1 and CRP4 completely lacked the 10×His-SUMO tag.

### 2.2. Secondary Structural Studies of Recombinant CRP1 and CRP4: The Impact of pH and Reducing Agents on Protein Folding

**Figure 4:**
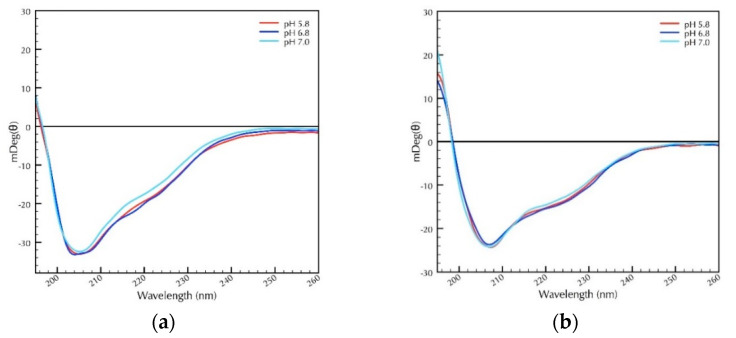

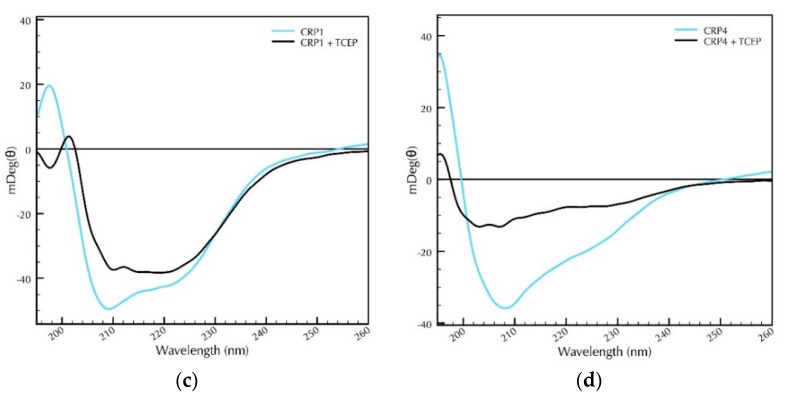
CD analyses of purified CRP1 and CRP4 under different pH conditions reveal large amounts of random coil and beta sheet conformation. Treatment of the recombinant proteins with the reducing agent TCEP resulted in changes in secondary structure. For pH studies, recombinant CRP1 or CRP4 were placed into different pH environments ranging from 5.8 to 7.0 and their secondary structural properties were investigated at 25 °C, while reducing studies were performed with TCEP at pH 7.0 and 25 °C. (**a**) CRP1; (**b**) CRP4; (**c**) CRP1 + TCEP; (**d**) CRP4 + TCEP.

After MS/MS analyses confirmed the successful purification of CRPs by chromatography, the recombinant proteins were subjected to CD spectroscopy to characterize secondary structural features. Prior to CD analysis, the homogeneity of the oxidation state of the CRPs was confirmed by SDS-PAGE analysis lacking reducing agent (Supplemental Figure S1). Structural conformations of CRP1 and CRP4 were analyzed under three distinct pH buffered potassium phosphate solutions, which included pH 5.8, 6.8, and 7.0 (Figure 4a,b). These pH conditions fall within proton concentrations reported within the ampulla of the MA gland and spinning duct of spiders [26]. Secondary structures were investigated by CD from 260 to 190 nm at 25 °C, a temperature that closely corresponds to physiological conditions of the silk-producing glands of black widow spiders [27].

At pH 7 the most abundant secondary structure of CRP1 was random coil conformation, with lower levels of beta sheet, beta turns, and alpha helical structure (Figure 4a; Table 1). These percentages corresponded to approximately 42% random coil, 35% beta sheet, 12% turns, and 10% alpha helices (Table 1). Under acidic or slightly acidic conditions of pH = 5.8 or 6.8, respectively, the secondary structure of CRP1 was not altered significantly (Figure 4a; Table 1). Analysis of the CRP4 CD spectra at pH 7.0, revealed approximately 45% random coil, 32% beta sheet, 11% beta turns, and 11% alpha helical structure (Figure 4b; Table 2). Similar conformations were observed at pH 5.8 and 6.8 (Figure 4b and Table 2). Comparatively speaking, the secondary structures of CRP1 and CRP4 at pH 5.8 to 7.0 generated similar profiles (Compare Figure 4a,b Table 1 and Table 2). Consistent with these experimental observations, the secondary structures of CRP1 and CRP4 closely align to predictions made by the NetSurfP 2.0 modeling algorithm (Table 1 and Table 2). Furthermore, extrapolation of the percent secondary structure contents from the modeled CRP1 three-dimensional structure using DSSP was also closely associated with the deconvoluted CD data (Table 1). 

To explore the supposition that CRP structure is dependent upon disulfide bonds, we treated the recombinant proteins with TCEP, followed by CD analysis to investigate whether reduction of cysteines within the protein chain impacted its structure. In both cases, treatment of the recombinant proteins with TCEP led to structural changes for both CRP1 and CRP4 at pH 7 (Figure 4c,d, respectively; Table 1, Table 2). Collectively, these findings, supporting CRP1 and CRP4, have substantial amounts of random coil conformation, followed by slightly lower levels of beta sheet structure. Overall, these studies also reveal little, if any, changes in the secondary structure across a pH range of 5.8 to 7.0; however, the conformations of the recombinant proteins were impacted by treatment with reducing agent, further supporting the assertion that disulfide bond linkages are important in the maintenance of secondary structure and that the bacterially purified recombinant proteins are indeed folded.

### 2.3. Secondary Structural Analysis of CRPs Under Different Temperature and pH

**Figure 5:**
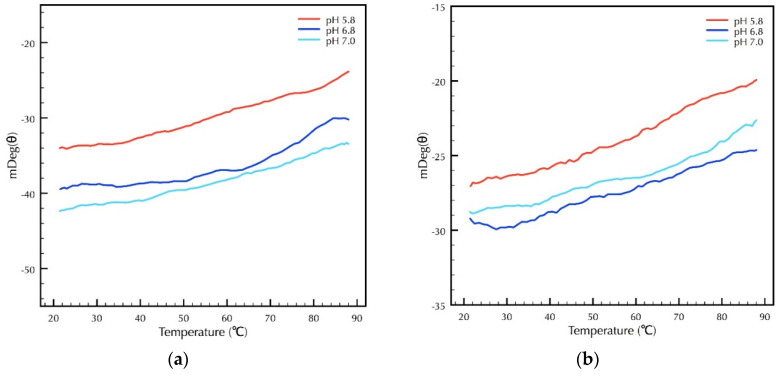
Thermal denaturation studies of CRPs under different pH conditions reveal proteins with high thermal stability. The millidegrees measured at 208 nm was monitored continuously in the temperature range from 20 to 90 °C by CD. Recombinant proteins were buffered at different pH of 5.8, 6.8, and 7.0. (**a**) CRP1; (**b**) CRP4.

After investigating the influence of pH on the secondary structure of CRP1 and CRP4 at 25 °C, we examined the effect of pH under different temperature conditions to explore thermal unfolding of the CRPs. Using CD spectroscopy, we generated thermal denaturation curves at 208 nm from 20 °C to 90 °C, a wavelength that corresponded to the minima of the secondary curves of CRP1 and CRP4 (Figure 4a,b), or over the entire far-UV range at various temperatures (Supplemental Figure S2). Within the investigated pH range of 5.8 to 7.0, the far-UV spectrum of native CRP1 and CRP4 revealed structures extremely resistant to unfolding at elevated temperatures, indicating enormous conformational stability up to 90 °C (Figure 5a,b).

Analysis of CD data at 90 °C suggests that CRPs have not yet approached their T_m_. Attempts to perform a protein thermal shift assay using GloMelt™ (Biotium, Fremont, CA, USA) to detect protein unfolding with fluorescence were unsuccessful, an observation that supports substantial amounts of hydrophobic residues are pre-exposed to the solvent while the CRPs are present within their native conformational state (Supplemental Figure S3). Thus, these studies support that CRP1 and CRP4 in their native states display both conformational stability from pH 5.8 to 7.0 and thermal tolerance from 20 to 90 °C.

### 2.4. XL-MS Studies Support Molecular Modeling of CRP1 as a Cystine Slipknot Member

**Figure 6:**
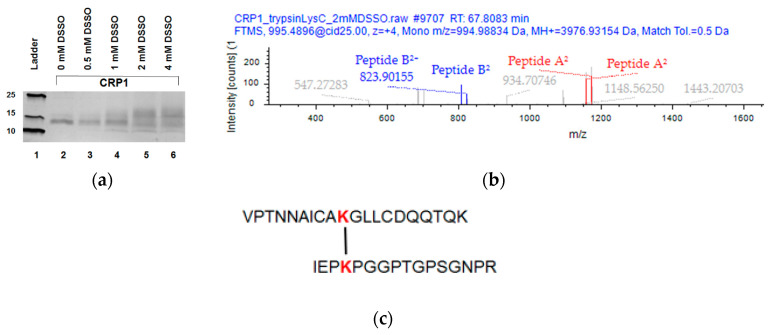
Cross-linking mass spectrometry (XL-MS) experiments with recombinant CRP1 treated with DSSO reveals one intramolecular cross-linked product between residues K53 and K75. (**a**) SDS-PAGE analysis of purified recombinant CRP1 treated with increasing concentrations of DSSO. Proteins were visualized by silver staining (**b**) MS/MS spectrum of the precursor ion containing the intramolecular cross-linked peptides (signature ion pairs are shown as red and blue lines). (**c**) CRP1 intramolecular DSSO-cross-linked peptides and their corresponding sequences.

According to published computational modeling of CRP1, lysine 53 (K53) and lysine 75 (K75) are in close structural proximity [23]. To further explore the validity of these models, we performed XL-MS with the cross-linking reagent DSSO and CRP1. Because this reagent contains N-hydroxysuccinimide (NHS) esters that react preferentially with K residues, we hypothesized that DSSO, which has a spacer length of 10.3 Å, should form covalent linkages between the primary amines of K53 and K75. To test this supposition, we incubated purified recombinant CRP1 with DSSO and examined the formation of cross-linked products by SDS-PAGE analysis, followed by visualization of proteins with silver staining (Figure 6a). DSSO-treated CRP1 led to the formation of slower migrating species relative to the non-cross-linked CRP1 control, supporting the formation of DSSO cross-linked products (Figure 6a, lanes 2–6). To determine the location of the cross-linking, we subjugated the 2 mM DSSO-treated CRP1 sample to in-solution LysC and tryptic digestion and analyzed the fragments by nLC-MS/MS. During MS analysis, a single intramolecular cross-linked peptide was identified by CID-MS/MS acquisition, yielding four signature fragment ions (Figure 6b). Signature ion pairs (two pairs or two peptides) were recognized by a mass difference of 31.9721 Da, triggering the signature fragment ions to undergo MS3 analysis, followed by their peptide identification using the XlinkX algorithm of Proteome Discoverer 2.4 (Thermo Fisher Scientific, Waltham, MA, USA). The cross-linked CRP1 product corresponded to IEPKPGGPTGPSGNPR and VPTNNAICAKGLLCDQQTQK, involving residues K53 and K75 with a XlinkX Score of 194.11 (Figure 6b,c).

**Figure 7:**
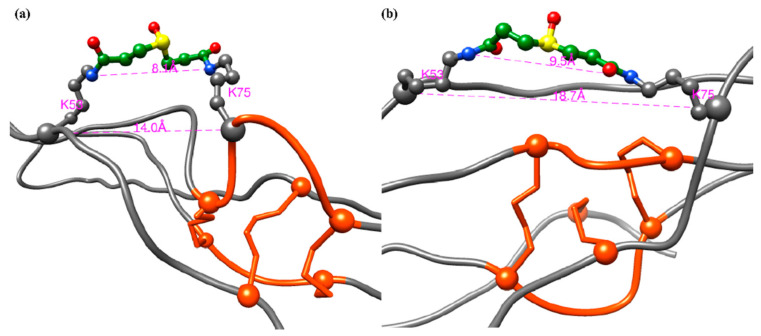
Ribbon diagram of CRP1 models cross-linked between K53 and K75 with DSSO, (**a**) model 1 and (**b**) model 2. The cysteines involved in cystine knots are shown as orange-red circles, and the Cα carbons are represented as spheres. Cross-linked K53 and K75 are shown in grey ball-and-stick representation. DSSO cross-linkers are shown in ball-and stick representation, where nitrogen atoms are in blue, carbon atoms are in green, oxygens are in red, and sulfurs are in yellow. The distance measurements (in Å) between Cα–Cα and N–N atoms of K53 and K75 residues are shown in magenta. Ribbon diagram of CRP1 models cross-linked between K53 and K75 with DSSO (**a**) model 1 and (**b**) model 2. The cysteines involved with cystine knots are shown as orange-red circles, and the Cα carbons are represented as spheres. Cross-linked K53 and K75 are shown in grey ball-and-stick representation. DSSO cross-linkers are shown in ball-and stick representation, where nitrogen atoms are in blue, carbon atoms are in green, oxygens are in red, and sulfurs are in yellow. The distance measurements (in Å) between Cα–Cα and N–N atoms of K53 and K75 residues are shown in magenta.

Based upon our XL-MS studies, our experimental data are consistent with both published models of CRP1 (Figure 7) [23]. Within the primary sequence of CRP1, there are three K residues, which include K53, K75, and K85. DSSO was capable of cross-linking K53 to K75, but K53 or K75 were not shown to cross-link to K85. Thus, given the DSSO spacer length of 10.3 Angstroms (Å), and the distance between Cα---Cα of K53 and K75 are 14 Å and 18.7 Å (models 1 and 2, respectively), our XL-MS data is consistent with the computational models of CRP1 and further substantiates the assertion of a cystine slipknot structure (Figure 7). To date, we have not built molecular models for CRP4, and given its primary sequence contains two K residues at K86 and K90, only four residues apart, it may not be ideal for DSSO cross-linking experiments.

## 3. Discussion

The molecular mechanisms that govern spidroin assembly and identification of constituents involved in the extrusion process are fundamental aspects to mimic synthetic silk perfection for commercialization. In our studies, we provide new insight regarding structural features for two CRP family members, demonstrating CRP1 and CRP4 have secondary structures that are largely random coil and beta sheet. Both recombinant proteins were expressed and purified from bacteria as 10xHis-SUMO-fusion proteins, followed by successful removal of the 10×His-SUMO tags through treatment with ULP1. Using purified CRP1 and CRP4 recombinant proteins, we show CRPs experience little, if any, structural transitions in response to pH changes, specifically hydrogen ion concentration from pH 5.8 to 7.0. Changes in pH have been reported to play a significant role in regulating spidroin assembly, in particular studies in orb-weavers have demonstrated that the spinning dope pH in the ampulla is 7.0, but drops to approximately 6.3 in the spinning duct [30,31]. This increase in acidity has been proposed to change the charge-state of specific R-groups on amino acids within spidroin protein chains, resulting in conformational changes, an event that helps trigger aggregation, assembly, and alignment of spidroins [11,32,33]. Both the N- and C-terminal domains (NTD and CTD) have been proposed to be important modulators of this process [11,34]. In fact, it has been reported that monomeric MaSp NTDs can dimerize when the pH changes from neutrality to acidic conditions [11]. This pH-regulated process has been implicated in triggering spidroin aggregation prior to extrusion [35].

Transcriptomic studies of the silk-producing glands from *L. hesperus* have detected CRP1, CRP2, and CRP3 transcripts in the MA gland [36]. However, based upon our proteomic studies we have detected CRP1, CRP2, and CRP4 in the MA gland, spinning dope, and dragline silk fiber [24]. This discrepancy may suggest other unknown environmental factors potentially allow for differential expression of CRP3 and CRP4. For example, the variable expression of MaSp1/2, along with AcSp1 in the MA gland of *L. hesperus*, might elicit expression of specific CRP family members. Initially, when we discovered the CRPs, we identified five different CRPs from a composite cDNA library constructed from all 7 silk-producing glands from *L. hesperus* [23]. Subsequent transcriptomic studies on *L. hesperus* largely support the aggregate gland expresses CRP5 in a tissue-restricted manner, while the pyriform gland expresses elevated levels of CRP2, CRP3, and CRP4, followed by the tubuliform gland predominantly expressing CRP1 mRNA [36]. In the minor ampullate (MI) and aciniform glands, little, if any CRP mRNA has been detected in *L. hesperus* [36]. Whether diet, geographic location, or age of *L. hesperus* plays a role in the expression of specific CRP family members remains unclear. Other spider species have been shown to express the CRPs, but there appears to be variable expression of specific CRPs in different silk glands, with the exception of CRP5, which largely exhibits an aggregrate gland-restricted pattern of expression across different species [36]. Interestingly, alignment of the amino acid sequences of CRP family members reveals CRP5 lacks a 14 amino acid internal segment within its protein chain relative to other CRP members, which could imply specific CRPs play differential roles during extrusion that are linked to fiber mechanics [23]. Further investigations to elucidate the relationship between the variable expression of CRPs in specific silk-producing glands and interactions with distinct spidroins, and their relevance to the mechanical properties of fibers, represent an exciting avenue to explore in the future.

Whether CRPs physically interact with spidroins is currently unclear; however, mechanistically speaking, our data support that acidification is unlikely to trigger CRP-conformational changes during extrusion of these proteins from the ampulla through the spinning duct, a central chemical event that induces spidroin assembly. In our current studies, the structural characteristics of CRP1 and CRP4 appear similar under different pH and temperature (non-reducing conditions); however, after CRP1 and CRP4 were treated with TCEP, their CD spectra were different, potentially because of differences between their amino acid sequences of their protein chains; the two proteins show 54% identities at the amino acid sequence level (Figure 1; secretion signal is lacking). Thus, more biochemical characterization will be necessary to elucidate the functional differences between CRP1 and CRP4, as well as more future studies to explore the structures of CRP2, CRP3, and CRP5.

Molecular modeling and sequence alignments with other proteins in the protein database suggest CRP members contain a cystine slipknot motif [23]. This structure is present within a fairly large number of secreted proteins and glycoproteins that belong to the TGFbeta and glycoprotein hormone (GPH) superfamily [37,38]. The cystine knot motif consists of three intertwined disulfide bonds. This structure has been described as one of the strongest force clamps known in nature, supported by molecular dynamics simulations [25]. Our current findings reveal the secondary structure of CRP1 and CRP4 can be impacted by treatment with TCEP, which supports that disulfide bonds play an important role in CRP structural maintenance. Furthermore, our CD spectroscopy and XL-MS studies buttress the assertion that CRP1 folds into a slipknot, which if assembled or interconnected with spidroins during extrusion could function as a nano-knot, serving to increase the extensibility and overall toughness of spider silk. Recent studies have been reported on the introduction of slipknots into Dyneema fibers, resulting in unprecedented toughness [39]. Whether variation of CRP expression helps modulate the formation of different numbers of knots or knot size in the fibers is intriguing, but further studies will be needed before a direct linkage between the CRPs and fiber mechanics can be firmly established. Our studies also reveal the CRPs also contain high thermal stability, which is also consistent with other cystine knot proteins, such as α-amylase inhibitor, who has a T_m_ over 100 °C [40,41]. In addition, other cystine knot-containing proteins are known to lack hydrophobic cores [40,41]. CRPs also lack hydrophobic cores, which is supported by our GloMelt™ thermal shift assay studies and NetSurfP-2.0 prediction program results. Furthermore, far-UV CD spectra reported from other cystine knot proteins closely resemble the CD spectra of the CRPs, implying structural similarities with established cystine knot proteins [41]. Overall, the biophysical properties of CRPs are consistent with reported cystine slipknot proteins and our findings support they serve a mechanical role in black widow spider threads.

## 4. Materials and Methods

### 4.1. Plasmid Construction and Expression of Recombinant CRP Proteins in Bacteria

CRP1 and CRP4 cDNAs were amplified by PCR and inserted into the prokaryotic pET19b-10×His-SUMO expression vector. CRP cDNAs were inserted in-frame into the expression vector, allowing for creation of N-terminal 10×His-SUMO tags on the CRPs. The pET19b-10×His-SUMO-CRP1 and pET19b-10×His-SUMO-CRP4 expression vectors were transformed into BL21(DE3)pLysS competent cells (Promega, Madison, WI, USA). Transformants were grown overnight and then used for protein inductions. After 4 h of induction at 37 °C, cells were pelleted and lysed in 1× FastBreak cell lysis reagent according to the manufacturer’s instructions (Promega, Madison, WI, USA). Extracts were sonicated 2 min to ensure complete bacterial cell lysis (10 sec pulses). Following sonication, crude extracts were clarified by centrifugation at 16,000× *g* for 10 min at 4 °C. Supernatants were filtered through a 0.2 micron filter and then used for affinity chromatography purification.

### 4.2. Purification of CRP1 and CRP4 using Ni-NTA Agarose Affinity Purification and Removal of the 10xHis-SUMO-tag

Recombinant 10×His-SUMO-CRP1 and CRP4 fusion proteins were purified using Ni-NTA affinity column chromatography. Bacterial supernatants were mixed with a Ni-NTA Superflow Agarose resin (Qiagen, Hilden, Germany), followed by incubation at 4 °C for 1 h with gentle agitation. After binding, the resin was washed two times with buffer (50 mM NaH_2_PO_4_, 300 mM NaCl, 20 mM imidazole, pH 8.0), followed by detachment of the recombinant CRP fusion proteins with elution buffer (50 mM NaH_2_PO_4_, 300 mM NaCl, 250 mM imidazole, pH 8.0). Following elution, imidazole was removed from purified samples using Spectra/Por^®^ Biotech cellulose dialysis tubing with a molecular weight cut off of 500–1000 Da (Sigma-Aldrich, St. Louis, MO, USA) by dialysis against a buffer (50 mM NaH_2_PO_4_, 300 mM NaCl, pH 8.0). 10×His-SUMO tags were removed from recombinant fusion proteins by treatment with purified His-tagged Ubiquitin-Like-Specific Protease 1 (ULP1) at 25 °C for 1 h. Following digestion, His-tagged ULP1 and 10×His-SUMO segments were removed by re-applying digestions to a Ni-NTA resin, where CRPs (lacking 10×His-SUMO) failed to interact with the resin and were present in the flow-through fraction. Then, non-tagged CRPs were concentrated and de-salted using Amicon^®^ Ultra-15 Centrifugal Filter Units with an Ultracel-3K membrane (EMD Millipore, Hayward, CA, USA) using a series of water washes. Protein concentrations were determined using a BCA protein assay kit according to the manufacturer’s instructions (Pierce Chemical Company, Rockford, IL, USA). Samples were size fractionated using sodium dodecyl sulfate polyacrylamide gel electrophoresis (SDS-PAGE) at 200V for 30 min. All samples for SDS-PAGE analysis contained 1× Laemmli buffer supplemented with 5% beta-mercaptoethanol and heated at 95 °C prior to electrophoresis. For protein detection, molecules were visualized with the ProteoSilver^™^ Silver Stain Kit according to the manufacturer’s instructions (Sigma-Aldrich). Images were captured on a ChemiDoc^™^ XRS+ system (Bio-Rad Laboratories, Hercules, CA, USA).

### 4.3. MS/MS Analysis Using a NanoLC Orbitrap Fusion™ Tribrid™ Mass Spectrometer

Tryptic digests of purified recombinant CRP1 and CRP4 (minus 10×His-SUMO tags) were analyzed by LC-MS/MS on a Thermo Scientific™ Orbitrap Fusion™ Tribrid Mass™ Spectrometer equipped with nanoHPLC coupled with an EASY-Spray™ ion source. Digests (5 µL or 0.25 µg) were injected onto a 20 mm × 75 µm C18 trap column (Thermo Scientific™) and run at a flow rate of 0.3 µL/min. The trap column was positioned in-line with the analytical EASY-Spray LC column (150 mm × 75 µm and particle size of 3 µm) for mass spectrometry analysis. Linear gradients of 1–50% solvent B over 33 min at 0.3 µL/min flow rate, followed by a steeper gradient from 50–99%, were used for peptide elution. Solvent B was held at 99% for 12 min to wash the column and returned to 1% solvent B. Before injection of the next sample, the column was washed by injecting water and running HCD fragmentation with the same chromatography method used for sample analysis. Solvent A and B consisted of 0.1% formic acid (aqueous) and 100% acetonitrile, respectively. The ionspray voltage was 1700 V, and interface heater was set at 300 °C. Full scan product ion data (MS^1^) were acquired using a data dependent acquisition mode with Xcalibur 4.0 software (Thermo Fisher Scientific, Waltham, MA, USA). Full scans were collected with the orbitrap mass analyzer using an AGC target of 1.0 × 10^6^ with a maximum inject time (quadrupole isolation window between *m/z* 300–1500 Da and injection of 100 ms) and resolution setting of 120,000. Peptide ions observed with orbitrap-MS scans exceeding a threshold of 100,000 and charge state of +2 to +8 were set to trigger acquisition of product ion fragmentation (quadrupole isolation window: *m/z* 1 Da, AGC target of 100,000, and injection time of 200 ms) where MS/MS spectra of the resultant 20 most intense ions were collected by HCD. The LC-MS/MS data were analyzed with Proteome Discoverer 2.4 (Thermo Fisher Scientific). The database search included an annotated Uniprot database for *L. hesperus* (downloaded March 17, 2016) containing all SwissProt and Trembl entries. In addition, a *L. hesperus* transcriptome was downloaded on July 29, 2016 from NCBI (BioProject PRJNA242358) and converted to an unannotated proteome fasta file using tools from galaxy.org [21]. A third custom database was manually made with known protein sequences that were not present in the Uniprot database, namely the sequence of CRP1. Precursor ions with a mass between 550 and 8500 Da were searched against the Uniprot database, using SEQUEST HT with a precursor mass tolerance of 10 ppm and fragment mass tolerances of 0.6 Da for HCD. Two fixed modifications were allowed, carbamidomethylation of cysteine and N-terminal acetylation, as well as the dynamic modification for oxidation of methionine.

For cross-linking mass spectrometry (XL-MS) studies, purified untagged CRP1 was subject to chemical cross-linking in a buffer containing 20 mM HEPES pH 7.8, 150 mM NaCl, and 1.5 mM MgCl_2_ supplemented with a protease inhibitor cocktail (cOmplete, Mini, EDTA-free tablets from Sigma-Aldrich). Cross-linking experiments were performed using DSS0 for 30 min at room temperature and then quenched with 20 mM Tris buffer pH 8. The optimal concentration of DSSO was determined by cross-linking recombinant CRP1 with a range of cross-linking reagent concentrations (0.5 mM to 4 mM) and visualization by SDS-PAGE and silver stain analysis. Cross-linked CRP1 molecules were digested with both LysC and trypsin, followed by desalting and peptide concentration and MS analysis using a recently published protocol [42,43].

### 4.4. Circular Dichroism Spectroscopy and DichroWeb

Purified protein samples were analyzed using a J-810 Spectropolarimeter with an MPTC-490S Auto Peltier 6-cell changer attached to a water pump temperature controller (Jasco, Inc., Easton, MD, USA.) using a quartz cuvette (Sigma-Aldrich, St. Louis, MO, USA) of 0.1 cm path length. For TCEP experiments, recombinant proteins were incubated with a final concentration of 5 mM. Data were collected using the SpectraManager software (Jasco, Inc., Easton, MD, USA), where measurements were taken with three accumulations from 260 to 190 nm with a data pitch of 0.5 nm and a scanning speed of 50 nm/min. Baseline measurements were taken using 10 mM potassium phosphate buffer at the desired pH. Data were exported to Microsoft Excel as xy-coordinates and graphed using Plot2. Thermal denaturation spectra were monitored at 208 nm from 20 °C to 90 °C at intervals of 0.5 °C using a heating ramp rate of 0.5 °C/min and temperature tolerance of 0.1 °C. Protein concentrations for CD analysis for CRP1 and CRP4 were 50 µM protein concentration. CD data were analyzed using DichroWeb (University of London, UK) to quantify the effects of pH on recombinant proteins. The Microsoft Excel sheet containing millidegrees data from 260 to 190 nm at a wavelength step of 0.5 nm was converted into a tab delimited text file and uploaded to DichroWeb under the “Free (with preview)” file format. Data were analyzed using the CDSSTR and CONTIN analysis programs coupled with the SMP180 reference set optimized for 190–240 nm. Structural values from both analysis programs were averaged. The mean residue weight (MRW) was calculated for both CRPs and the cell path length used was 0.1 cm.

## Figures and Tables

**Table 1:** Secondary structure of CRP1 broken down into the four main types of secondary structure identified by DichroWeb. * Predicted percentages were calculated from the 8-state protein secondary structure (SS_8_) predicted model produced by NetSurfP-2.0, with bend collapsed into random coil structure. Percentages of secondary structures of the modeled CRP1 three-dimensional structure were calculated using the DSSP algorithm [28,29].

Percentage of Each Characteristic at 25 °C for CRP1				
	Random Coil	Beta Sheet	Turns	Helix
pH 5.8	45.6	31.35	11.95	10.6
pH 6.8	45.1	31.1	11.95	11.4
pH 7.0	41.85	35.25	11.75	10.15
pH 7.0 + TCEP	37.9	33.8	15.2	12.6
Predicted *	43.5	30.7	18.2	7.9
DSSP algorithm	44.4	23.9	21.6	10.2

**Table 2:** Secondary Structure of CRP4 broken down into the four main types of secondary structure identified by DichroWeb. * Predicted percentages were calculated from the 8-state protein secondary structure (SS_8_) predicted model produced by NetSurfP-2.0, with bend collapsed into random coil structure.

Percentage of Each Characteristic at 25 °C for CRP4				
	Random Coil	Beta Sheet	Turns	Helix
pH 5.8	44.95	33	11.25	10.3
pH 6.8	42.85	34.3	11.65	10.65
pH 7.0	45	32.35	11.15	11.05
pH 7.0 + TCEP	38.8	43.2	13.3	3.7
Predicted *	45.5	33.3	17.8	3.3

## Supplementary Materials

The following are available online, Figure S1: SDS-PAGE analysis of purified recombinant CRP1 lacking the SUMO tag (-SUMO) under non-reducing conditions; Figure S2: Far-UV spectra for purified CRP1 (-SUMO tag) at different temperatures reveals the thermal stability of CRP1; Figure S3: In its native state CRP4 has a substantial amount of hydrophobic residues that are exposed to solvent.
