# Supplementary material for: Structural Characterization of Black Widow Spider Dragline Silk Proteins CRP1 and CRP4
Source: Molecules. 2020 Jul 14;25(14):3212. doi: 10.3390/molecules25143212 (PMC7397007; doi:10.3390/molecules25143212)
Supplement: Supplementary file 1 [file molecules-25-03212-s001.pdf]

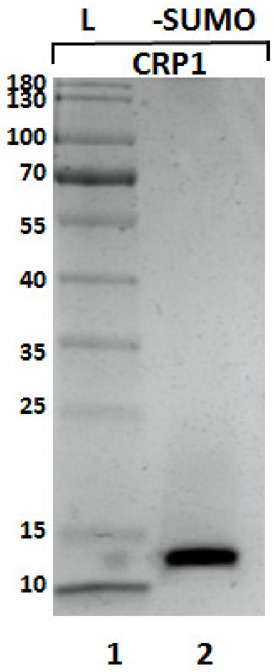

Figure 1S. SDS-PAGE analysis of purified recombinant CRP1 lacking the SUMO tag (–SUMO) under non-reducing conditions. Proteins were visualized by silver stain analysis. Lane 1 represents a protein ladder (L) in kDa, while lane 2 contains CRP1 with no reducing agent.

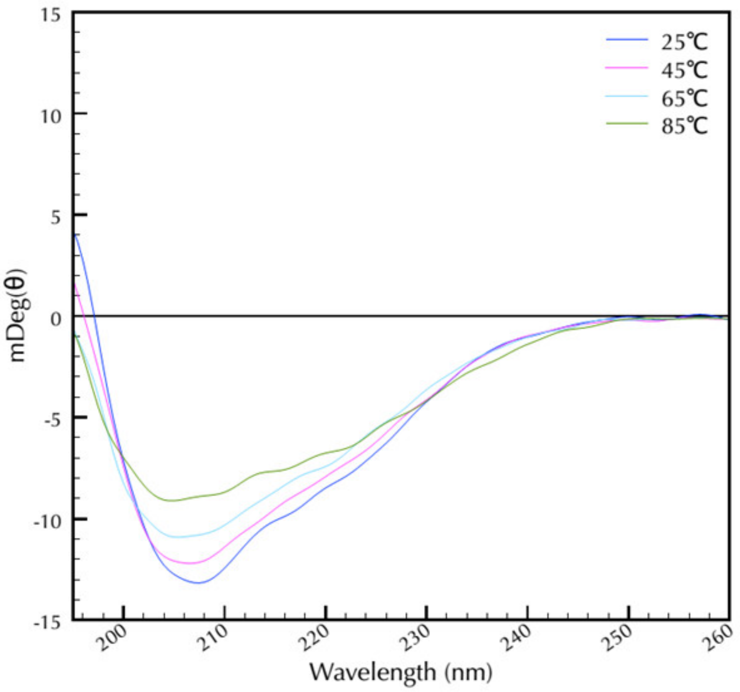

Figure 2S. Far-UV CD spectra for purified CRP1 (-SUMO tag) at different temperatures (25, 45, 65 and 85 degree Celsius at pH = 5.8) reveals the thermal stability of CRP1. The analysis was performed at 25 µM and deconvolution of the spectra revealed similar secondary structure contents.

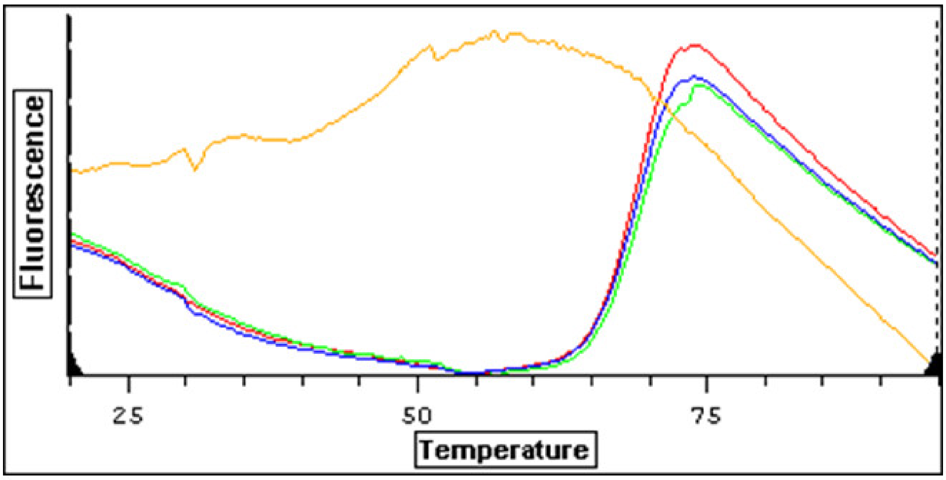

Figure 3S. In its native state CRP4 has a substantial amount of hydrophobic residues that are exposed to solvent. CRP4 (gold) and IgG (control; red, blue, and green) were analyzed using the GloMelt Thermal Shift Protein Stability kit according to the manufacturer's instructions (Biotium). Similar results were observed for CRP1.
